# The Ultimate Results Obtained in the Open-Air Treatment of Pulmonary Tuberculosis

**Published:** 1903-10-31

**Authors:** H. Hyslop Thomson

**Affiliations:** Physician to the Consumption Sanatorium of Scotland, Bridge of Weir.


### Hospital Clinics and Medical Progress.

### THE ULTIMATE RESULTS OBTAINED IN THE OPEN-AIR TREATMENT OF PULMONARY TUBERCULOSIS.

By H. Hyslop Thomson, M.D., Physician to the Consumption Sanatorium of Scotland, Bridge of Weir.

During recent years much has been written on the treatment of pulmonary tuberculosis by the open-air method, and although it is now generally accepted that it gives better results than any hitherto obtained by other methods, there still exists some degree of scepticism as to its value. In view of this it is obviously desirable that the subsequent history of those who have been resident in a sanatorium should be carefully followed up, so that information may be forthcoming as to the real value of the treatment, and to what extent, and for what period of time those who have benefited by it remain in good health and are able to work. At the outset it must be emphasised that the open-air treatment is not a specific—it merely aims at conserving the natural resisting and recuperative powers of the tissues; moreover, it must be continued in a modified form as a precautionary measure, hence we must not expect it to prove successful in all cases, and disappointing results have to be faced. Stated generally, the good results which follow the sanatorium treatment of consumption are as follows :—

1. In a certain proportion of cases, when the subsequent conditions are favourable, the disease is permanently arrested.
2. In a further proportion of cases the disease is temporarily arrested, and the life of the wage-earner prolonged.
3. For a certain period of time the isolation of an infectious disease is obtained.
4. During the residence in a sanatorium instruction is given in the methods to be adopted to prevent the spread of consumption.

The almost immediate effect of pure air on some of the more distressing symptoms of pulmonary tuberculosis is striking. It speedily soothes the cough and diminishes the amount of expectoration, thereby contributing much to the comfort of the patient. At the same time it stimulates the appetite, checks night sweating, and lowers the fever which invariably accompanies the disease, the natural result being that emaciation is arrested, the general health improves, and the body weight increases by anything from a few ounces to several pounds per week. Unfortunately, however, the physical signs do not become modified *pari passu* with the amelioration of the local and general symptoms, but if the treatment be persevered with the physical evidence of active disease becomes less marked and the morbid process gradually becomes arrested, or may indeed finally be obliterated by the formation of healthy scar tissue.

The rapidity and the extent of the response to treatment varies greatly in different individuals, success or failure depending mainly upon three factors.

1. The stage in the course of the disease at which treatment is commenced.
2. The intensity of the infective process, that is to say whether the condition is one of chronic, subacute or acute phthisis.
3. The degree of resistance, and the recuperative powers possessed by the patient.

Now while it can be positively stated that the immediate results which follow the open-air treatment of pulmonary tuberculosis are good, one is constrained to speak with less enthusiasm of the ultimate results obtained, although these, as indicated by the figures given below, are quite sufficient to stamp this method as one of unprecedented value.

In tabulating results the term "cure" has been frequently applied to those cases in which the disease has become arrested, but we must admit that in this relationship the term is somewhat misleading. The word in itself implies the impossibility of any relapse, and relapse we know is but too common even in cases which have been most favourably influenced by treatment. The reasons for this are obvious. It is well known that the tubercle bacilli may exist in a quiescent nodule in one or other lung and give rise to no marked symptoms either local or general, and few physical signs; in such cases the danger of relapse is always imminent. And even if the morbid process is obliterated by scar tissue free from tubercle bacilli, there still exists the danger of recurrence from reinfection, owing to the enfeebled resistance of the pulmonary tissue. Lastly, unhealthy surroundings, poor living, and negligence in carrying out preventive measures are important factors in favouring relapse from either cause.

In dealing with this subject, I have not endeavoured in any way to magnify the good results obtained, as it is very desirable that any estimate formed as to the value of the open-air method of treating tuberculosis should be based on carefully-collected statistics bearing on the condition of health, not less than two to three years after treatment.

The subjoined figures indicate the number of patients discharged from the Bridge-of-Weir Sanatorium as "well" or "practically well" during the three years 1898–1900, and their condition in the year 1903. In all 142 patients were discharged during that time, of whom 56 were returned as "well" or "practically well." The following are the particulars of their subsequent history :—

(*a*) Of 9 patients discharged in 1898-1899—

| | |
|---|---|
| Well or fairly well and able to work... | 3 |
| Fairly well, but not able to work ... | 1 |
| Lost sight of ... ... ... ... | 2 |
| Dead ... ... ... ... ... | 3 |
| Total ... ... ... | 9 |

(*b*) Of 24 patients discharged in 1899–1900—

| | |
|---|---|
| Well or fairly well and able to work... | 13 |
| Lost sight of ... ... ... ... | 1 |
| Dead ... ... ... ... ... | 10 |
| Total ... ... ... | 24 |

(*c*) Of 23 patients discharged in latter part of 1900—

| | |
|---|---|
| Well or fairly well and able to work... | 21 |
| Dead ... ... ... ... ... | 2 |
| Total ... ... ... | 23 |

Regarding the cases in which death has taken place, the pulmonary disease, so far as is known, was the direct cause in 10; in one case death followed childbirth, while in the remaining 4 laryngeal tuberculosis, kidney disease, abdominal tuberculosis, and general tuberculous lymphadenitis were present.

Of the 38 cases at present alive the diagnosis of pulmonary tuberculosis was confirmed by the detection of tubercle bacilli in the sputum of 27, and in 11 the diagnosis was based on the presence of physical signs associated with hæmoptysis or evening pyrexia.

The stage in the course of the disease at which treatment was commenced in the 38 cases mentioned above was as follows:—

| Early. | Intermediate. | Advanced. |
|---|---|---|
| 26 | 9 | 3 |

The following are the particulars regarding the duties or employment in which they are at present engaged:—

| | |
|---|---|
| Married, and attending to household duties ... ... ... ... | 17 |
| Doing housework ... ... ... | 7 |
| Domestics ... ... ... ... | 3 |
| Factory-workers ... ... ... | 3 |
| Dressmakers ... ... ... ... | 2 |
| Needleworkers... ... ... ... | 2 |
| Governess ... ... ... ... | 1 |
| Book-keeper ... ... ... ... | 1 |
| Machinist ... ... ... ... | 1 |
| At home ... ... ... ... | 1 |
| Total ... ... | 38 |

To sum up, the figures given above show that of the total number of patients discharged during the three years 1898–1900, 39·4 per cent. were returned as "well" or "practically well," and that of that number 67·8 per cent. are alive and in good or fair health after a lapse of three years. It should be mentioned, however, that these figures do not include all who are at present alive and well. A further percentage were discharged as "very much improved," and although the writer has made no systematic inquiry regarding their subsequent history, a certain number are known to be alive and in good health.

In conclusion, we have formed the opinion from these statistics that, although the open-air treatment of tuberculosis may not be all we could desire, it is surely of sufficient value to convince the most sceptical that it is indeed worthy of trial, and that, until a better method of treating this disease be discovered, it is the only one likely to prolong life and appreciably diminish the prevalence of tuberculosis.

